# Surgically generated aerosol and mitigation strategies: combined use of irrigation, respirators and suction massively reduces particulate matter aerosol

**DOI:** 10.1007/s00701-021-04874-4

**Published:** 2021-05-24

**Authors:** Moritz W. J. Schramm, Asim J. Sheikh, Rebecca Chave-Cox, James McQuaid, Rachel C. W. Whitty, Evgenia Ilyinskaya

**Affiliations:** 1 Department of Neurosciences, The General Infirmary At Leeds, Great George Street, Leeds, LS1 3EX UK; 2 School of Earth and Environment, University of Leeds, Leeds, UK

**Keywords:** Aerosol, Surgical smoke, Mitigation, Filtration

## Abstract

**Background:**

Aerosol is a health risk to theatre staff. This laboratory study quantifies the reduction in particulate matter aerosol concentrations produced by electrocautery and drilling when using mitigation strategies such as irrigation, respirator filtration and suction in a lab environment to prepare for future work under live OR conditions.

**Methods:**

We combined one aerosol-generating procedure (monopolar cutting or coagulating diathermy or high-speed diamond- or steel-tipped drilling of cadaveric porcine tissue) with one or multiple mitigation strategies (instrument irrigation, plume suction and filtration using an FFP3 respirator filter) and using an optical particle counter to measure particulate matter aerosol size and concentrations.

**Results:**

Significant aerosol concentrations were observed during all aerosol-generating procedures with concentrations exceeding 3 × 10^6^ particles per 100 ml. Considerable reductions in concentrations were observed with mitigation. In drilling, suction, FFP3 filtration and wash alone respectively reduced aerosol by 19.3–31.6%, 65.1–70.8% and 97.2 to > 99.9%. The greatest reduction (97.38 to > 99.9%) was observed when combining irrigation and filtration. Coagulating diathermy reduced concentrations by 88.0–96.6% relative to cutting, but produced larger particles. Suction alone, and suction with filtration reduced aerosol concentration by 41.0–49.6% and 88.9–97.4% respectively. No tested mitigation strategies returned aerosol concentrations to baseline.

**Conclusion:**

Aerosol concentrations are significantly reduced through the combined use of filtration, suction and irrigation. Further research is required to characterise aerosol concentrations in the live OR and to find acceptable exposure limits, and in their absence, to find methods to further reduce exposure to theatre staff.

## Introduction

Surgeons and theatre staff are routinely exposed to a variety of occupational hazards. Air pollution is one such hazard. Staff may be exposed to a variety of pollutants, which include aerosol particulate matter (PM): microscopic particulates suspended in the air. A variety of surgical instruments are known to generate PM, amongst the most commonly used in neurosurgery are electrocautery and high-speed drills.

Inhalation of PM poses health hazards in its own right: various negative health effects (mainly respiratory and cardiovascular diseases leading to reduction in life expectancy) associated with exposure to elevated airborne PM concentrations, indoors and outdoors, are now well recognised [14, 15]. Size of the PM is an important factor for health impacts, as finer particulates can penetrate deeper when respired. Air quality guidelines have been established by World Health Organisation for long-term exposure (annual and 24-h) to coarse particulates PM10 (fraction of PM ≤ 10 µm diameter) and fine particulates PM2.5 (≤ 2.5 µm diameter). Both epidemiological and clinical studies have also demonstrated that sub-daily exposures to elevated concentrations of PM—common for workplace exposures—can lead to adverse physiological changes in the respiratory and cardiovascular systems, but no guidelines for short-term exposure limits have been established [3, 23]. In addition to the potential for direct harm from PM, there is the potential for transmission of live pathogens through PM aerosol [5, 25]. The advent of the SARS-CoV 2 outbreak has brought PM to the attention of the surgical community as a potential vector for the transmission of this virus. The virus appears to remain viable for at least 3 h in aerosol [9]. Indoor PM aerosol has been proposed as one key mechanism in facilitating the spread of SARS-CoV 2 [16], and has been heavily implicated in individual superspreader events [19]. Surgically generated PM is considered by some as a potential vector for transmission, although this remains disputed. Neurosurgeons are at risk of accidental or deliberate exposure to PM generated from respiratory epithelium, for instance during endonasal or translabyrinthine procedures, or through encountering the frontal air sinus. Presence in brain tissue is likely much less [21], if at all, but the virus appears to be able to infect choroid plexus [22]. A large number of policies have been implemented at various institutions to manage the hitherto difficult-to-quantify risk of transmission from patient to theatre staff.

The most commonly used neurosurgical instruments known to generate PM aerosol are electrosurgical instruments and high-speed drills. In electrosurgery, radiofrequency currents at very high frequencies are used to heat tissue. Electrosurgical instruments have been shown to generate significant particulate matter aerosol, and authors have identified a number of factors that affect how much, including the type of tissue being worked on [13], the type of instrument used [12] and theatre conditions with the airflow in theatres being important, both in terms of the use of laminar flow theatres reducing aerosol burden [11] and in terms of airflow can be altered by the micro-environment e.g. the heat generated by surgical lamps, personnel and other instruments [2].

Respiratory hazards in surgical smoke have been recognised and studied in the past; however, we found few reports on mitigation strategies for the theatre staff’s exposure to respiratory pollutants [4, 13, 18, 25]. Notable examples of mitigation strategy testing included a study testing N95 respirators [10], a study investigating surgery inside a negative-pressure aerosol containment chamber [8]. Anecdotally, it has been suggested that surgeons ought to use suction to reduce the air pollution burden experience by theatre staff, and irrigation has been proffered by some as a valid method for reducing PM aerosol generation during drilling. In the present study, we use an optical particle measurement technique with high time-resolution to demonstrate significant reductions in aerosol particle concentration when using a variety of mitigation strategies either on their own or in combination. We trial the use of suction and FFP3 respirators to reduce aerosol concentration in drilling and electrocautery, as well as the use of wash in drilling.

## Materials and methods

### Particulate matter measurements

All PM aerosol measurements were obtained in the same unventilated room using a 16-channel optical particle counter (OPC), GRIMM Portable Laser Aerosolspectrometer and Dust Monitor Model 1.108, [GRIMM Aerosol Technik GmbH & Co. KG, Ainring, Germany] with a sampling interval of 6 s. The size range of particulate matter measured was from 0.3 to 20 µm. The choice of sampling interval and particle size range was the maximal settings supported by the instrument. Twenty micrometres is significant as representing the size at which less than 10% of the inhaled particles are part of what the European Committee for Standardisation consider the thoracic fraction, namely, particles that penetrate the airway beyond the larynx [28], and none penetrate to the unciliated airways. The sampling inlet of the OPC was extended using low-friction tubing to sample from roughly 40 cm from the working site. Forty centimetres was chosen as it corresponds to roughly the middle of the range of average working distances in surgery e.g. when sizing surgical loupes [1], and thus gives a reasonable approximation of where the surgeon’s face would be relative to the aerosol-generating instrument. The room’s baseline PM number concentrations were measured for a total of 1 h. In order to obtain these reference data, equipment was set up on an experimental day and recording begun prior to any drilling or electrocautery.

### PM aerosol-generating procedures

PM aerosol was generated using a high-speed drill and monopolar electrosurgical cautery. Drilling was done by hand with a Stryker Sumex drill running at 75,000 rpm with either a 4 mm 2-fluted stainless steel ‘Precision Round’ burr or a 4 mm fine diamond burr [Stryker UK, Newbury, United Kingdom] on the cortex of cadaveric porcine long bone. Monopolar cautery was conducted on muscle tissue in cadaveric porcine abdominal wall specimens using an ERBE VIO 200 D [Erbe Medical UK Ltd., Leeds, United Kingdom] with Cutting set to Auto Cut Mode with max. Watts set at 80 W; coagulation settings were Forced Coag Mode with maximal watts set to 80 W. All runs were conducted by the same experimenter.

### PM aerosol mitigating interventions

We here refer to experimental conditions as combinations of any one PM aerosol-generating procedure with none, one or several PM aerosol mitigating interventions. PM aerosol mitigating interventions were smoke extraction, irrigation and filtration. Smoke was extracted using a portable suction machine [QuickClear Rescue, GBUK Healthcare, Selby, United Kingdom] set to maximal suction. Irrigation for drilling was provided with 0.9% saline solution coaxially mounted to the drill. Filtration was obtained by attaching an EN 149-compliant [29] FFP3 filtering face piece mask [Easimask FSM16, Full Support Group, Wellingborough, United Kingdom] to the sampling inlet. The experimenter was using a full face respirator with ABEK1P3 filters [Moldex Metric, Walddorfhäslach, Germany].

For each experimental condition, just over 1 min of continuous activity was carried out at a time. The particle measurements from the 60 s during which the aerosol-generating procedure was carried out was analysed. A total of 5 repeats of each experimental condition were carried out. Prior to commencing the next experimental run, PM levels were allowed to return to the room’s baseline. The time necessary for this varied between 10 and 30 min, depending on the PM concentration at the end of the aerosol-generating procedure and decay followed an exponential function. The 10 measurements for each of the 5 × 60 s runs were pooled and analysed together. For the purpose of comparison, 50 consecutive baseline measurements were pseudo-randomly chosen from the whole baseline dataset.

Data are reported as mean and mean differences with 95% confidence intervals. No null-hypothesis significance testing was conducted.

## Results

**Fig. 1:**
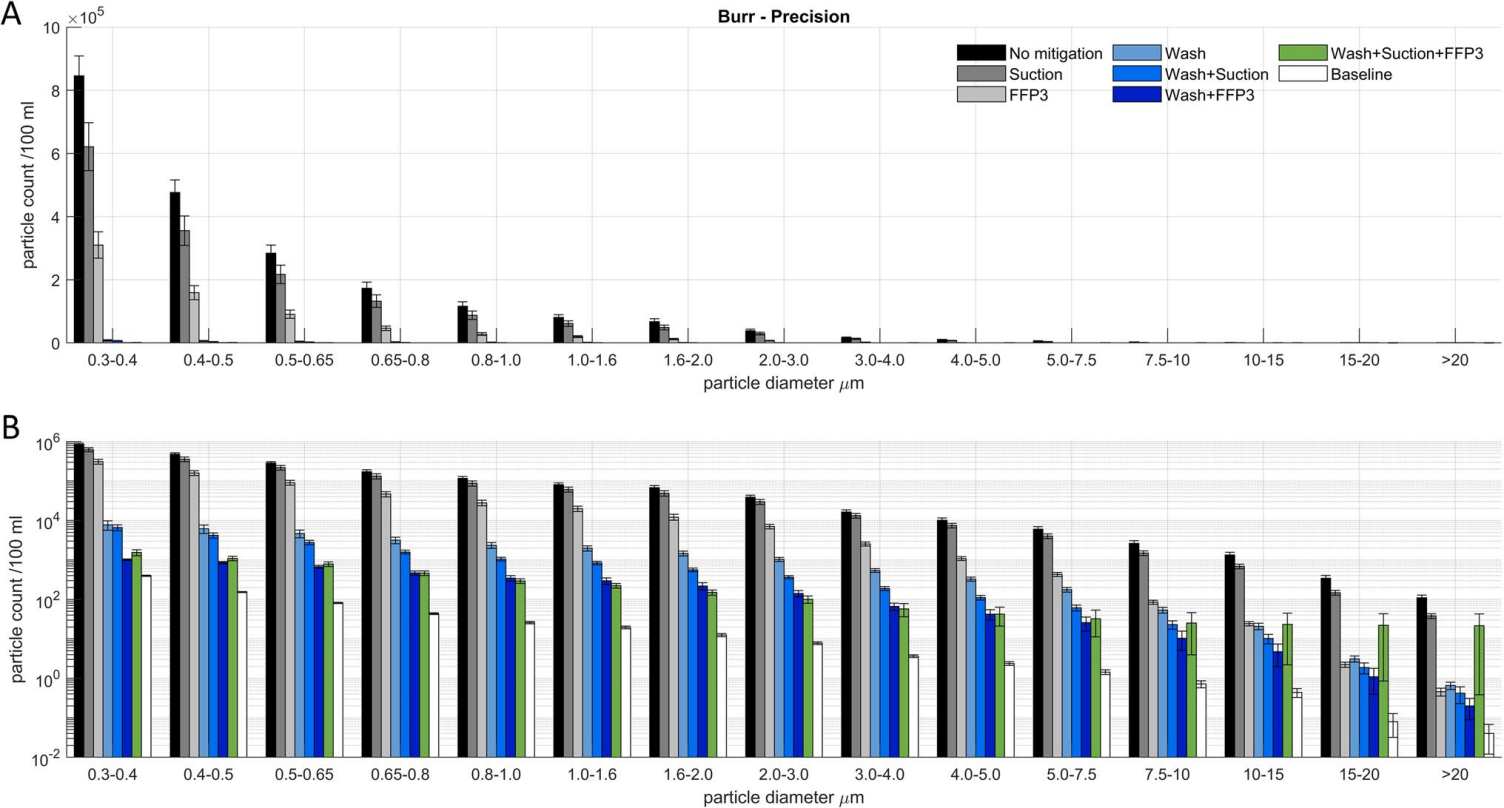
Aerosol particle size distribution for experimental condition using the ‘Precision’ Burr, plotted with a (**a**) standard scale and a (**b**) logarithmic scale. Error bars represent 1 S.E.M. Particle counts are per 100 ml. Particle sizes are reported as diameter (µm)

**Fig. 2:**
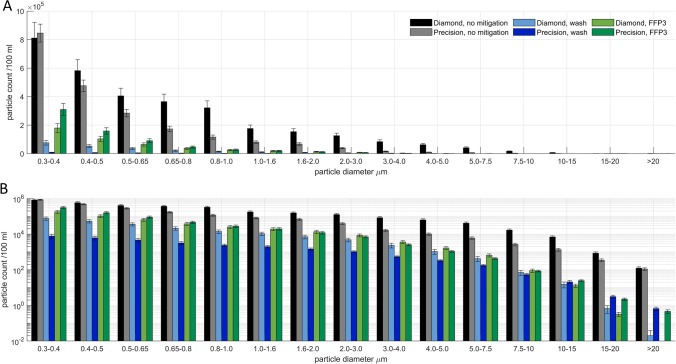
Aerosol particle size distribution for experimental condition using the ‘Diamond’ Burr comparing with equivalent conditions obtained using the ‘Precision’ burr, plotted with a (**a**) standard scale and a (**b**) logarithmic scale. Error bars represent 1 S.E.M. Particle counts are per 100 ml. Particle sizes are reported as diameter (µm)

**Fig. 3:**
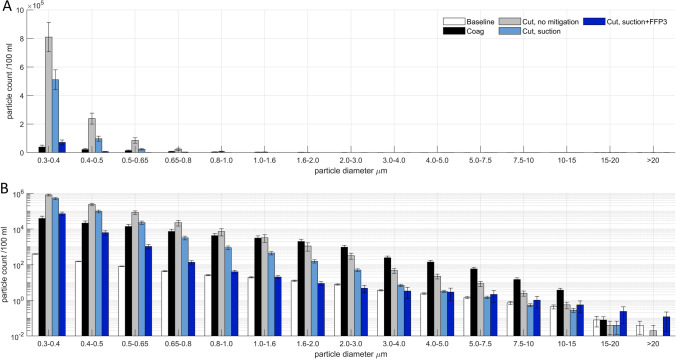
Aerosol particle size distribution for experimental condition using cutting and coagulating monopolar electrocautery, plotted with a (**a**) standard scale and a (**b**) logarithmic scale. Error bars represent 1 S.E.M. Particle counts are per 100 ml. Particle sizes are reported as diameter (µm)

All experimental conditions generated particulate concentrations significantly above baseline. Room baseline PM number concentrations averaged 7 × 10^2^ particles / 100 ml. Unmitigated drilling and electrocautery produced mean PM number concentrations in the order of between 1–2 × 10^6^ particles / 100 ml for drilling with 2-fluted and diamond burrs, respectively, and 1 × 10^5^ to 1 × 10^6^ particles / 100 ml for monopolar electrocautery in coagulation and cutting mode, respectively. Particle sizes followed an approximately logarithmic distribution, both at room baseline and during aerosol-generating procedures. The vast majority of observed particles were ultrafine, 1 µm or less in diameter (PM1), accounting for between 80 and 99% of the total PM number concentration across experimental conditions. Cutting monopolar cautery appeared to have a particular predominance in producing PM1, with PM1 accounting for > 99% of observed particulate matter across all experimental conditions—the particle size distributions are shown in Figs. 1, 2 and 3.

For drilling with a steel 2-fluted ‘precision’ burr, all of the tested mitigation strategies appeared to reduce observed aerosol concentrations to some extent, the single greatest effect was observed when using wash: the introduction of wash alone reduced total PM concentrations from 2.12 × 10^6^ (± 0.2 × 10^6^) to 3.0 × 10^4^ (± 0.6 × 10^4^) particles / 100 ml, a reduction by 70-fold. When combined with the use of suction and an FFP3 respirator, PM1 concentrations fell further to 4.9 × 10^3^ (± 0.8 × 10^3^) particles / 100 ml, only about 1 order of magnitude off baseline or a 430-fold reduction to 0.2% of the unmitigated aerosol concentration. Similarly, when using a diamond burr, unmitigated average total PM concentrations were 3.2 × 10^6^ (± 0.4 × 10^6^) particles / 100 ml—in excess of the upper bound of the instrument’s dynamic range—falling to 4.6 × 10^5^ (± 0.8 × 10^5^) particles / 100 ml when using an FFP3 respirator and 2.3 × 10^5^ (± 0.5 × 10^6^) particles / 100 ml when using wash.

When using electrocautery, total PM concentrations of 1.2 × 10^6^ (± 0.2 × 10^6^) particles / 100 ml when using unmitigated cutting monopolar diathermy and 9.2 × 10^4^ (± 2.9 × 10^4^) particles / 100 ml when using coagulating monopolar diathermy were observed. Cutting diathermy was mitigated to 6.4 × 10^5^ (± 0.9 × 10^5^) particles / 100 ml when adding suction, and to 7.9 × 10^4^ (± 1.9 × 10^4^) particles / 100 ml when combining suction and use of an FFP3 respirator, for a reduction to less than 7% of the unmitigated aerosol burden.

## Discussion

This study was designed as synthetic benchmark for aerosol generation. The design principles were twofold: firstly, to minimise the impact of outside variables such as the complex airflow of live operating theatres [2], the different tissues being worked on in real surgery and the changing of instrument settings. For this reason, we chose to conduct the study in cadaveric tissue with fixed instrument settings and in a room without ventilation. Secondly, we aimed to maximise aerosol generation to be able to better demonstrate and estimate the effect size of mitigation strategies. We effectively use an in vitro study that faithfully recreates the use of PM aerosol-generating surgical instruments on tissue and we quantify PM aerosol concentrations using an optical particle counter, allowing direct, near-real time quantification of airborne particles and stratification by particle size. The key limitations of our study are that it does not recreate atmospheric conditions inside an operating theatre nor aims to measure the real-world efficacy of the tested mask, but rather give an assessment of the maximal effect size obtainable by using mitigation strategies when excluding precisely these variables. With these caveats, the results presented in this report once again confirm, in keeping with the existing literature, that both high-speed drills and electrocautery have the potential to generate very significant amounts of PM aerosol and that relatively simple manoeuvres could massively reduce the amount of PM aerosol that operating staff are exposed to, albeit not to baseline.

To our knowledge, the present report is the first published dataset quantifying the extent to which the simple, intraoperative mitigation strategies of irrigation and suction can reduce the theatre aerosol burden alone or in combination. We found that combining multiple strategies is greatly reduces PM aerosol burden: we found that through the combination of wash, irrigation and an FFP3 respirator, the aerosol concentration generated by drilling can be reduced from millions to thousands of particles, a 400-fold reduction to less than a fraction of a percent of the unmitigated aerosol concentration. The single biggest contributor to this reduction appeared to be the use of wash, with the use of an FFP3 respirator providing a modest reduction. Suction appeared to add little when FFP3 respirators and wash are combined, but did appear to provide a modest benefit in itself or in combination with only irrigation or an FFP3 respirator. In electrocautery, where wash is not readily deliverable without compromising instrument performance, the maximal reduction achieved by combining the use of an FFP3 respirator and suction was from reducing the particulate matter concentration from millions of particles to tens of thousands. Furthermore, using coagulating as opposed to cutting monopolar electrocautery also resulted in a considerable change in PM aerosol composition—fewer, but larger particles were seen in coagulating diathermy, whereas cutting diathermy produced the opposite distribution.

An interesting observation is the effect FFP3 respirators had on particle burden in our study. FFP3 respirators are standardised according to European norms which put them approximately on par with the US N99 respirator standard in terms of filtering PM aerosol [7]. These respirators are frequently cited in the media and marketing material as filtering in excess of 99% of particulate matter. This is likely in reference to the filter efficiency, which for P3 filters is 99.05% when tested with sodium chloride and paraffin PM aerosol [29]. It is worth noting that, in the UK, the assigned protection factor (APF) cited for FFP3 masks is 20 while the nominal protection factor (NPF) is 50 [7], closer to the reduction in particle counts we observed but still substantially greater (Table 1). The NPF is derived from laboratory studies measuring total inward leakage of a test particulate matter when the respirator is worn. The APF is the factor by which a respirator is expected to protect workers in real-life conditions. The APF is the reference factor to estimate the real-world protection expected from a given respirator while the NPF represents a best-case laboratory scenario. The difference between NPF and the filter efficiency is down to the filter efficiency describing the theoretical filtering properties of the filter material used in perfect laboratory conditions with no leak (which in the case of an FFP3 respirator should indeed filter upward of 99% of sub 0.6 micron particles) whereas the NPF tests the respirator as a system, including its face seal, albeit under idealised, standardised conditions relative to the workplace. It should therefore be self-evident that our data cannot be understood as an assessment of NPF or APF as the respirator filter material was attached to the air intake of our instrument, rather than worn by a participant. With that proviso, the performance we observed was significantly worse yet than both NPF and APF, with a 5- to tenfold reduction in particulate matter concentrations observed at best. It is therefore likely that the apparent discrepancy in the present data between what users might expect and the reduction in particulate matter we observed is down to leakage, which fits with no marked change in aerosol size distribution when comparing aerosol filtered through an FFP3 respirator with unmitigated aerosol—if the filter’s maximal filtration was the limiting factor, one would expect particles of about 0.6 µm diameter to be the most likely to penetrate through the filter, as this is viewed as the particle size most likely to penetrate by the CEN due to the particles’ unpredictable movement patterns, varying between Brownian motion and laminar flow [20, 29].

Interestingly, Elmashae et al. found significantly higher attenuation of particle number concentrations when using N95 respirators, ranging from reducing particle counts by a factor of just below ten to nearly 1000, averaging a factor of about 153. This is a significantly greater attenuation than the cited APF for N95 respirators, which is expected to be 10 according to the Occupational Health and Safety Administration [7]. There are significant differences between the methodologies of the study presented here and Elmashae’s work, most notably in that Elmashae et al. included much smaller particles in their counts, used respirators of a different standard and, crucially, had human participants wearing the respirators. In their study, particulate matter number concentrations were measured simultaneously outside and inside the respirator, a design for measuring the real-world effectiveness of the mask in protecting a human wearing a respirator. It is also worth noting that reports exist that employ a similar methodology to Elmashae et al., but find protection factors to be significantly less than expected [17], and further reports find that the expected efficacy of the filter material (excluding factors such as leakage) may be less than expected when testing for aerosols other than the charge-neutralised monodisperse aerosol used in the EN149 guideline [26]. Therefore, a likely explanation for the worse-than-expected performance of FFP3 masks in the experiments presented herein is substandard seal around the respirator mount; however, given the limited data available on the performance of particulate respirators for reducing particulate matter surgical aerosol, further work is needed.

**Table 1:** Observed particle counts in various experimental conditions and observed absolute and relative reduction upon introduction of mitigating strategies

Initial condition	Initial value (particle count /100 ml)	Comparator	PM_total_ absolute Reduction seen (95% CI)	95% CI of %age reduction from Initial
Diamond Burr, no mitigation	3.15 × 10^6^	Diamond + wash	2.93 × 10^6^ ± (0.12 × 10^6^)	89.2–96.8%
		Diamond + FFP3	2.69 × 10^6^ ± (0.12 × 10^6^)	81.6–89.2%
		Precision, no mitigation	1.03 × 10^6^ (0.12 × 10^6^)	28.9–36.5%
Precision Burr, no mitigation	2.12 × 10^6^	Precision + wash	2.09 × 10^6^ ± (0.03 × 10^6^)	97.2 to > 99.9%
		Precision + FFP3	1.44 × 10^6^ ± (0.06 × 10^6^)	65.1–70.8%
		Precision + suction	0.54 × 10^6^ ± (0.13 × 10^6^)	19.3–31.6%
Precision Burr, + wash	2.97 × 10^4^	Precision + wash + FFP3	2.55 × 10^4^ ± (0.17 × 10^4^)	80.1–91.6%
		Precision + wash + suction	1.13 × 10^4^ ± (0.18 × 10^4^)	31.9–44.1%
Precision Burr, + wash + FFP3	4.16 × 10^3^	Precision + wash + FFP3 + suction	− 7.21 × 10^2^ ± (2.56 × 10^2^)	− 23.5 to − 11.1%
Diamond Burr + wash	2.27 × 10^5^	Vs Precision Burr + wash	1.97 × 10^5^ ± (0.13 × 10^6^)	81.1–92.5%
Cutting Monopolar	1.17 × 10^6^	Cutting Monopolar + suction	0.53 × 10^6^ ± (0.05 × 10^6^)	41.0–49.6%
		Cutting Monopolar + suction + FFP3	1.09 × 10^6^ ± (0.05 × 10^6^)	88.9–97.4%
		Vs Coag Mono	1.08 × 10^6^ ± (0.05 × 10^6^)	88.0–96.6%
Cutting Monopolar + Suction	0.64 × 10^6^	Cutting Monopolar + suction + FFP3	0.56 × 10^6^ ± (0.03 × 10^6^)	82.8–92.2%

Similarly, a study by Workman et al. [27] showed quite a significant reduction in particle count by several orders of magnitude in prolonged drilling (60 s and more) when using suction in the context of endonasal drilling. However, in this setting, the travel of particulate matter aerosol is limited by the enclosed space within which work takes place and as such, the micro-environment is more readily controlled by suction which may account for the different outcomes. A few authors have proposed the use of barrier methods [6, 8] to protect from patient-to-staff intraoperative transmission of SARS-CoV2, again showing significant reductions in what the authors term aerosol and droplet contamination. In both studies, contamination of the surgical field was studied using fluorescent dye and visual or camera inspection of surfaces. These methods do not measure airborne particulate matter, but rather deposited droplets. Furthermore, Chen et al. state their method cannot detect particles smaller than 100 μm. Given that the patient-generated particles smaller than 5 μm have been shown to carry intact and replicating SARS-CoV2 virions [24], this method of sampling would appear insufficiently sensitive to demonstrate an absence of spread beyond the confines of the barrier.

We caution the reader to not view the figures presented herein as an estimate of theatre staff’s exposure to aerosol in a live OR, even when using the exact same instruments and settings as used in these experiments for several reasons. As previously stated, this experiment was designed to deliberately exclude some of the factors that would affect aerosol concentrations in real-world operating theatres and may introduce unanticipated variation into our data. Furthermore, it is worth pointing out that our suction system was not a dedicated smoke extraction system but a simple suction unit. We also cannot readily extrapolate from our data of drilling on cortical long bone to how mucosa-covered bone may behave—in our experiments, residual soft-tissue could temporarily reduce aerosol generation.

With regards to respiratory protection, two important comments need to be made: the first observation concerns the health-risk of PM aerosol in itself, and is that if relying on FFP3 filtration alone to mitigate surgical aerosol, assuming they perform at their assigned protection factor, a 20-fold reduction will likely provide only partial protection from the aerosol burden given the very high concentrations seen in this study, and for this purpose, adding irrigation and/or suction would be prudent. The second observation concerns the potential for PM aerosol as a vector of disease transmission from patient to theatre staff. While from a general health perspective, lower particle concentrations are likely to be better for health, we would expressly caution against simply assuming that lower particulate matter concentrations provide surgical staff with better protection from contracting the SARS-CoV2 or other live pathogens–while this may be true, it is hypothetically possible that an intervention was done in good faith to reduce exposure to live virus paradoxically increases the risk. For instance, it is not inconceivable that wash reduces the heat generated during drilling and that the heat generated during drilling of, say, peri-nasal bone damages virus particles to the point of non-viability, and that the reduced temperatures and lower aerosol burden means that even though there are fewer total particles, the particles that are generated contain more viable virions. Based on our findings, the aerosol burden theatre staff are exposed to is likely to be considerable and probably constitutes a health risk, even in the absence of the potential for viral transmission, given the emerging findings of the wider air quality research community. Theatre staff ought to be protected from this health risk. A successful mitigation strategy against an occupational hazard relies on: knowing the extent of the hazard, knowing the possible mitigation strategies and the quantitative effect they have on the extent of the hazard, and knowing a reasonable limit of exposure to the hazard that can be tolerated. We would argue that the key addition we have made towards a successful mitigation strategy is furthering our understanding of the possible mitigation strategies. We show that any one individual strategy has a limited impact, but multiple strategies tend to complement each other. The key gaps we identify all concern themselves with safe limits. As explained above, without knowing the limits on exposure to PM aerosol, it is impossible to write guidance beyond recommending the principle of maximum precaution.

We believe that our data should serve as a call to action to better characterise surgical staff’s exposure to aerosol and devise mitigation strategies. We identify the following gaps in the literature:

1. What are the exposure limits for surgically generated aerosol?
2. What are the exposure limits for surgically generated aerosol that may contain live pathogen? How do particulate matter sizes affect the risk of transmission from patient to staff during an operation? How do mitigation strategies affect the amount of viable virus that reach the surgical staff?
3. What factors determine the amount and size distribution of surgically generated aerosol, and what can surgical teams do to mitigate? To what extent do factors such as temperature, tissue composition, instrument material composition and others play a role?

In summary, drilling and monopolar electrocautery produce significant PM aerosol concentrations. These can be mitigated, but only partially, with filtration, suction and (where suitable) irrigation. Considerable work is required to determine what level of protection is required for surgeons and must differentiate between the health effect of PM aerosol itself, and the potential for PM aerosol to act as a vector for pathogens. Different strategies may be required to counter these two potential pathways to harm.

## Abbreviations

FFP3: A European respirator standard: Filtering Facepiece respirator, P3, see EN149 [29]
ml: Millilitre
N95/N99: A US filtration standard: N95/99 Particulate Filter (95/99% filter efficiency level) not resistant to oil. Frequently used synonymously with respirators utilising the filter class
PM(x): Particulate matter, where (*x*), if given, denotes the maximal size of particle, in micrometer, included in the classification (PM5, particulate matter no larger than 5 μm)
rpm: Revolutions per minute
W: Watt
